# Monitoring of Epoxy-Grouted Bonding Strength Development between an Anchored Steel Bar and Concrete Using PZT-Enabled Active Sensing

**DOI:** 10.3390/s19092096

**Published:** 2019-05-06

**Authors:** Jian Jiang, Chuang Hei, Qian Feng, Jinwei Jiang

**Affiliations:** 1 Key Laboratory of Earthquake Geodesy, Institute of Seismology, China Earthquake Administration, Wuhan 430071, China; jiangjian@eqhb.gov.cn; 2 Wuhan Institute of Earthquake Engineering Co., Ltd., Wuhan 430071, China; 3 School of Electronics and Information, Yangtze University, Jingzhou 434023, China; heichuang@yangtzeu.edu.cn; 4 Department of Mechanical Engineering, University of Houston, Houston, TX 77204, USA

**Keywords:** anchored steel bar, lead zirconate titanate (PZT) transducer, bonding strength development monitoring, active sensing

## Abstract

Anchored steel bars have been widely used in retrofitting of existing concrete structures. The bonding strength between the anchored steel bar and the concrete is critical to the integrity of the strengthened concrete structure. This paper presents a method to monitor epoxy-grouted bonding strength development by using a piezoceramic-enabled active sensing technique. One concrete beam with an anchored steel bar was involved in the monitoring test, and two concrete beams with six anchored steel bars were used in the pull-out test. To enable the active sensing, a Lead Zirconate Titanate (PZT) patch was bonded to the surface of the exposed end, and piezoceramic smart aggregates were embedded in each concrete specimen. During the monitoring experiment, signals from PZT sensors and smart aggregates were acquired at intervals of 0, 20, 40, 60, 80, and 100 min. In addition, a pull-out test was performed on each of the remaining six anchored steel bars in the two concrete beams, while the signal was recorded in the test. Furthermore, a wavelet packet analysis was applied to analyze the received signal energies to investigate the bonding strength development between the concrete and the anchored steel bar during the epoxy solidification process. The test results demonstrate the effectiveness of the proposed method in monitoring the bonding strength development between the anchored steel bar and the concrete, using the PZT-enabled active sensing.

## 1. Introduction

The retrofit, reinforcement, and strengthening of existing structures have been widely practiced and researched [1,2], and various guidelines and standards have been developed [3,4,5,6]. Anchored steel bar technology has been widely used in retrofits and new construction since the 1970s [7]. The technology, as a post-installed connection method, plants a steel bar into a concrete base with structural adhesives [8]. Anchored steel bars in concrete are required when an additional structural member is to be linked to the existing concrete structure [9]. Integrated with the original structural members, the anchored steel bars make the entire structural system more reliable, increasing the bearing capacity of the structure [10].

Due to its wide application, the anchored steel bar technology has received much attention in the literature. In the 1990s, Cook [11] and Cook et al. [12,13] carried out a series of experimental studies on bonded anchors and adhesive anchors. The result showed that the bonding strength could fully meet the requirements of structural bearing capacity when the anchoring and bonding measures were sufficient. Furthermore, Cook and Konz [14] investigated various factors that influence the bond strength of polymer-based adhesive anchors. Li et al. [15] addressed the failure mechanism and the failure load of quadruple fastenings with bonded anchors. The authors proposed a model for evaluating the failure load of quadruple fastenings with bonded anchors based on the numerical and experimental results. Yilmaz et al. [16] and Çalışkan et al. [17] studied the tensile behavior and shear strength of epoxy anchors embedded into low-strength concrete. Barnaf et al. [18] focused on bond strength experiments using high-strength concrete up to class C50/60 or higher. Assaad and Issa [19] studied the bond strength of epoxy-coated bars in underwater concrete. Smith et al. [1] developed analytical models for anchored bars with experimental verification.

Presently, epoxy is one of the commonly used adhesives in the anchored steel bar technology. In addition, the epoxy can repair concrete cracks to prevent or reduce further corrosion [20,21,22,23]. For the sake of achieving the bonding strength required for a project, it is necessary to mix the two different components of the epoxy in a certain proportion to produce a chemical reaction. A pull-out test for steel bars is typically conducted to detect the bonding strength between steel bars and concrete [24,25]. This method is destructive. Sakla and Ashour [26] used artificial neural networks (ANNs) to estimate the tensile capacity of single adhesive anchors.

Piezoceramic materials, exhibiting piezoelectricity, have been one of the popular materials used in structural health monitoring [27,28,29,30] due to their low cost, fast response [31], embeddability [32,33,34,35], and dual ability of actuation and sensing [36,37,38,39]. Furthermore, because of its strong piezoelectric effect [40,41] and wide bandwidth [42,43,44], Lead Zirconate Titanate (PZT) is one of the most commonly used piezoceramic materials, and is frequently utilized for stress wave generation [45,46,47,48] and detection [49,50,51]. Stress waves are often used in structural health monitoring [52,53,54,55,56], especially in the active sensing method [57,58,59,60,61] and the electromechanical impedance method [62,63,64,65,66]. The active sensing method was used for monitoring bolt loosening [58], very early age cement hydration [59], a bolted spherical joint connection [60], and timber moisture [61]. The impedance method was used for monitoring the damage in plate-like structures [62], bolted joint looseness [63], pin connection loosening [64], the freeze–thaw process in soil [65], and a concrete-filled fiber-reinforced polymer tube [66]. The PZT-enabled active sensing approach using surface-mounted or embedded transducers has shown great potential for the structural health monitoring of mechanical and civil structures in real time [67,68,69,70].

The PZT-enabled active sensing method has been found to be effective in detecting de-bonding between a steel plate and concrete [71,72,73], an FRP (fiber-reinforced polymer) bar and concrete [74,75,76], and other structural components [77,78,79]. However, few studies have been reported on using this method to monitor the bonding strength development of an anchored steel bar and concrete by using epoxy, to the authors’ best knowledge. This motivated the authors to conduct a feasibility study of real-time monitoring of the epoxy-grouted bonding strength development between a steel bar and the host concrete structure. To demonstrate the effectiveness of the proposed method, a concrete specimen with a drilled hole, involved with two embedded PZT-based smart aggregates and two surface-bonded PZT patches, was used. An anchored steel bar was grouted to the concrete specimen through the drilled hole with epoxy. The epoxy develops its full bonding strength within 80–100 min at room temperature. A PZT patch was additionally mounted on the exposed end of the steel bar. The active sensing experiments were conducted for 100 min. The experimental results clearly show that the PZT sensor signals increase with the time, i.e., increase with the bonding strength of the epoxy, demonstrating the feasibility of monitoring bonding strength between the steel bar and the concrete by using PZT-enabled active sensing.

## 2. Principles

### 2.1. Piezoceramic-Based Active Sensing Approach

**Figure 1:**
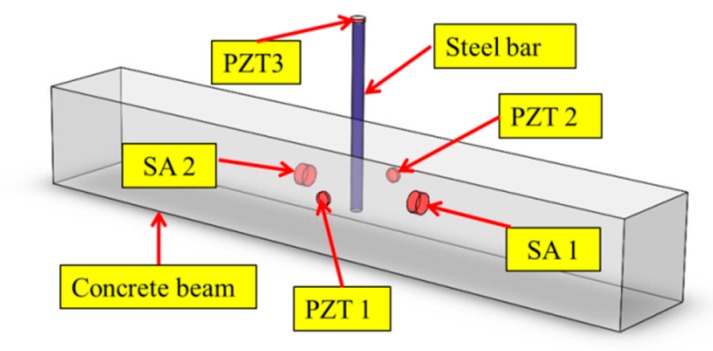
Sketch of the specimen with deployed SAs (lead zirconate titanate smart aggregates) and PZT (lead zirconate titanate) transducers.

Due to the piezoelectricity, PZT can be either used as an actuator or a sensor [36,39,46]. Therefore, PZT transducers and PZT smart aggregates can detect the propagating stress wave when they are deployed on the wave path [48,55,60]. Figure 1 shows a three-dimensional (3D) sketch of the specimen. Two PZT transducers, namely PZT1 and PZT2, were mounted to the concrete beam surface as sensors. Two PZT smart aggregates (SAs), namely SA1 and SA2, were embedded in the concrete beam by drilling holes in the existing concrete. One PZT transducer, namely PZT3, was mounted on the steel bar as an actuator. In this research, the specimen with PZT3 attached to the exposed end of the steel bar was used to verify the feasibility of monitoring epoxy-grouted bonding strength by using the PZT-enabled active sensing technique. 

**Figure 2:**
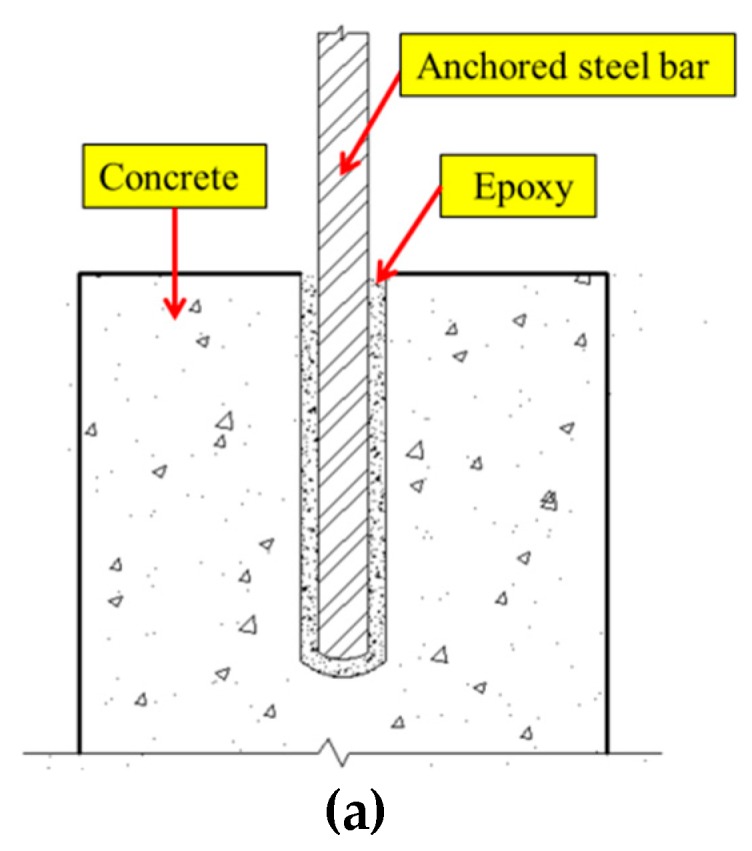

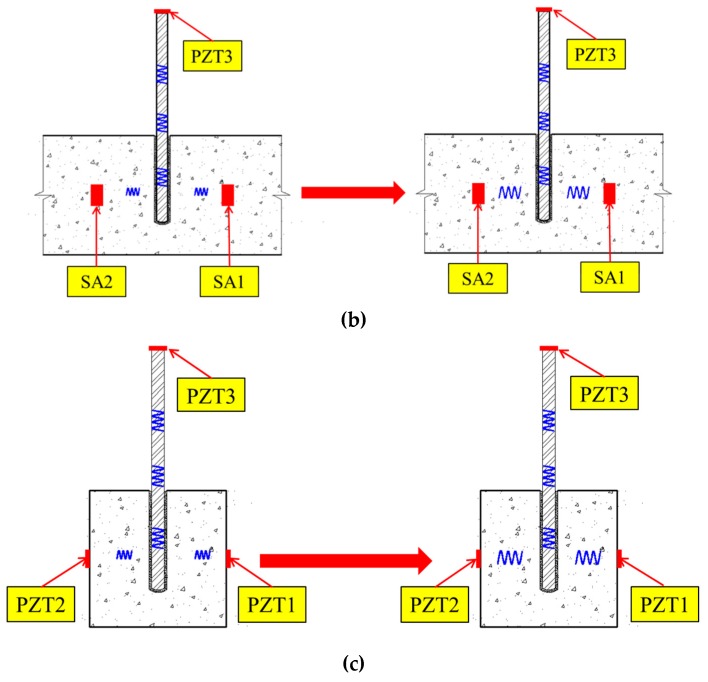
Stress-wave-based active sensing principle for bonding strength monitoring: (**a**) Details of the anchored steel bar in concrete with epoxy; (**b**) Received signals of SA1 and SA2 during the epoxy solidification process; (**c**) Received signals of PZT1 and PZT2 during the epoxy solidification process.

Figure 2 shows the schematic of the active sensing approach in the monitoring of the epoxy-grouted bonding strength development between the anchored steel bar and the concrete during the epoxy solidification process. The stress wave generated by PZT3 propagates from the anchored steel bar to the concrete beam through the epoxy inside the hole. PZT1 and PZT2 are on the concrete beam’s surface, and SA1 and SA2 are inside of the concrete beam. Therefore, these transducers can detect the stress wave, since the concrete material is a good conduit for stress wave propagation [68,71,75]. During the curing process of the epoxy, the propagation of stress waves highly depends on the status of the transmission medium [80]. When the epoxy is in the liquid form, the compressive stress wave highly attenuates and the corresponding detected signals are very weak [59,81,82]. When the epoxy cures, the increased stiffness of the epoxy will directly influence the characteristics of the received signal from PZT3. Simultaneously, the epoxy-grouted bonding strength between the anchored steel bar and the concrete beam increases, and the stress wave energy propagated from the steel bar to the concrete will be greatly strengthened. As a result, the signal strength received by PZT1, PZT2, SA1, and SA2 will also increase, respectively. Based on these signals, the strength development of epoxy between the anchored steel bar and the concrete can be monitored.

**Remark:**  In some applications, the exposed end of the steel bar may not be available to be bonded with the PZT patch. In such cases, we will design and try re-usable PZT transducers with integrated magnets. These re-usable PZT transducers can be attached to the end surface or the side surface of the anchored steel rod by using magnetic force. Furthermore, the re-usable PZT transducers will help to broaden the potential applications of the proposed approach.

### 2.2. Wavelet-Packet-Based Energy Index

The wavelet-packet-based energy index (WPEI) analysis has been demonstrated to be an effective technique to quantitatively estimate the differences between the stress wave responses due to structural changes, such as damage [22,46,60,67,68,72,73], and structural material phase changes, such as concrete curing [59,80] and soil freeze–thaw [81]. In wavelet analysis, a signal can be divided into several wavelet packets and the signal energy of each packet can be computed [56,67]. Consequently, the energy of the signal can be computed by the summation of energies of all the packets [67,79]. The received signal is decomposed by n-level wavelet packet decomposition into $2^{n}$ signal sets $\left\{X_{1},X_{2},\ldots ,X_{2^{n}}\right\}$ in each decomposed signal from the original signal $X_{j}$**,** where j is the frequency band $j=1,2,\ldots ,2^{n}$. In this study, n=5. $X_{j}$ can be further expressed as:

(1) $$X_{j}=\left[x_{j,1},x_{j,2},\ldots ,x_{j,m}\right]$$

where m is the sampling data collected from the data acquisition system. Thus, the energy of the decomposed signal $E_{j}$ can be computed as:

(2) $$E_{j}=\overset{k=m}{\underset{k=1}{\sum }}x_{j,k}^{2}$$

The total energy of the signal can be computed by the summation of all the decomposed signals. The computed energy of the signal E can be expressed as:

(3) $$E=\overset{2^{n}}{\underset{j=1}{\sum }}E_{j\;}$$

The received signal can be characterized by using the WPEI analysis. The WPEI has been used to monitor or evaluate the structural health condition in various concrete structure applications [82,83]. In this research, the WPEI was applied to monitor the received signal energy change in the active sensing approach when the bonding strength between the concrete and the steel bar was changing.

## 3. Experimental Setup and Procedure

### 3.1. Specimen Fabrication

**Figure 3:**
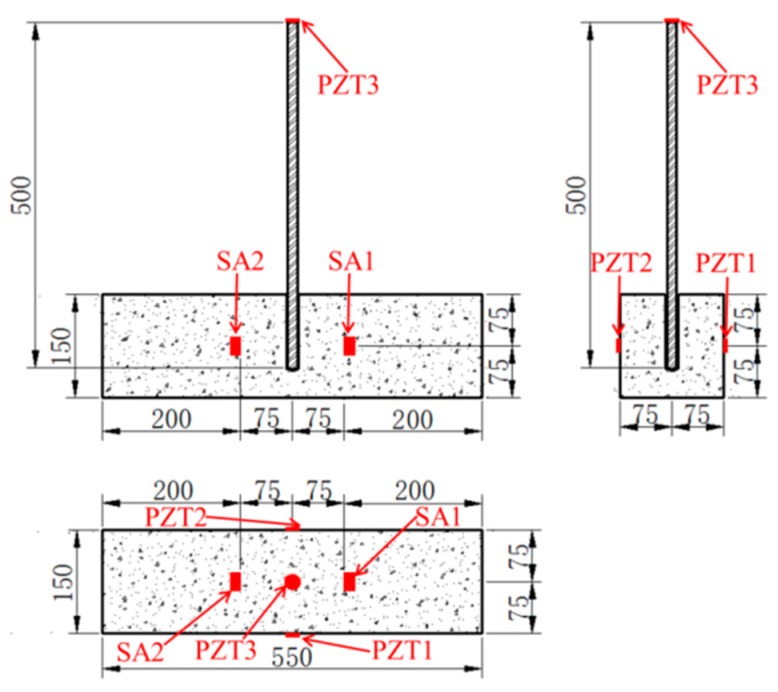
Dimensions of the specimen and the location of PZTs and SAs.

**Figure 4:**
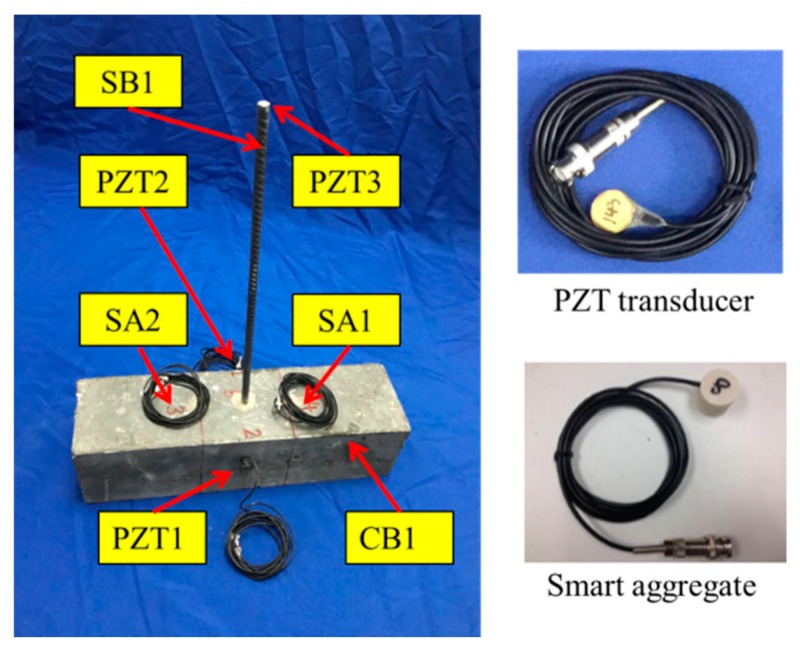
A specimen with an anchored steel bar.

**Figure 5:**
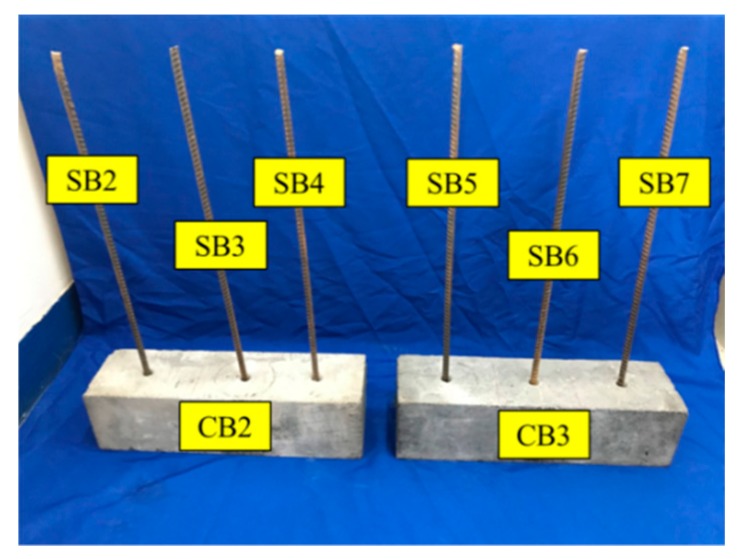
Specimens with anchored steel bars for the pull-out test.

The dimensions of the specimen for the monitoring test and the location of PZTs and SAs are presented in Figure 3. PZT1 and PZT2 were attached to the concrete beam (CB1) surface by epoxy, while SA1 and SA2 were embedded into CB1 by drilling holes in the concrete. Additionally, PZT3 was bonded to the surface of the exposed end of the steel bar (SB1) by epoxy. The locations of the three PZT transducers and the two smart aggregates are shown in Figure 3. Figure 4 shows the experimental specimen for the monitoring test. In order to find out the connection between the energy index and bonding strength, it is necessary to perform the monitoring test and the pull-out test simultaneously. Figure 5 shows the experimental specimen for the pull-out test. There are two concrete beams (CB2 and CB3) and three steel bars (SB2–SB7) were anchored into each of them, while SB1 was anchored into CB1 with the same epoxy.

The dimensions of concrete beams CB1–CB3 is 550 mm × 150 mm × 150 mm and each specimen was made with concrete of class C40. The size of steel bars SB1–SB7 is Φ12 mm × 50 mm and the type is HRB335. The material properties of the test specimens are shown in Table 1. The depth of all the anchored steel bars within the concrete beam is 120 mm.

### 3.2. Experimental Setup

**Figure 6:**
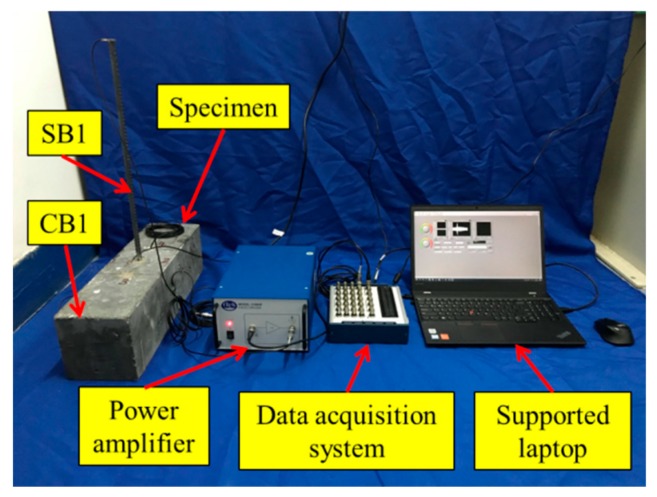
Experimental setup of the monitoring test.

The experimental setup of the monitoring test includes the concrete beam (CB1) with an anchored steel bar (SB1), PZTs and SAs, a power amplifier (Trek 2100HF) for piezoceramic load, and a data acquisition system (NI USB6363) hosted by a laptop computer, as shown in Figure 6. The sampling frequency of the data acquisition system was 2 MS/s. During the test, a sweep sine wave was generated by the data acquisition system, and then amplified by the power amplifier with a gain of 50. The amplified signal was used to drive the actuated signals of PZT3. PZT1, PZT2, SA1, and SA2 were used as sensors to receive signals from the concrete. Signals were recorded at the 0, 20, 40, 60, 80, and 100 min mark during this experiment. The start frequency, stop frequency, amplitude, and time interval of the excitation signal were 1000 Hz, 150 kHz, 10 V, and 0.5 s, respectively.

**Figure 7:**
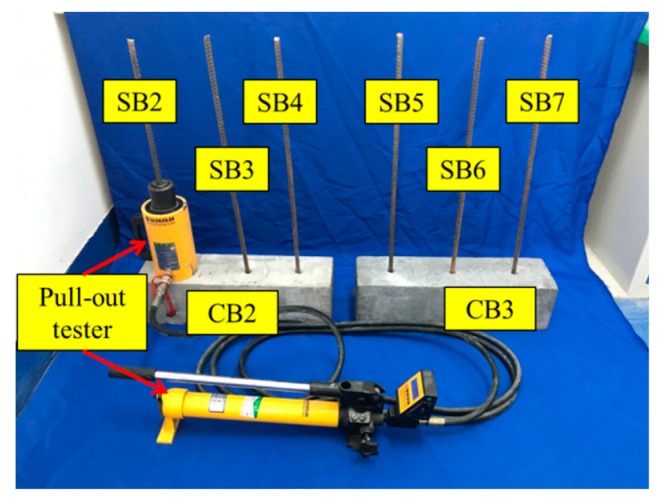
Experimental setup of the pull-out test.

The pull-out test involved two concrete beams (CB2 and CB3) with anchored steel bars (SB2–SB7), as shown in Figure 7. A hydraulic pump was used to apply the load during the pull-out test. SB2, SB3, SB4, SB5, SB6, and SB7 were pulled out at the 0, 20, 40, 60, 80, and 100 min mark, respectively. The ambient temperature was approximately 15 ℃ during the experiments. Since all the tests were conducted within 2 h in the laboratory, the influence of temperature disturbance on the results was negligible.

## 4. Experimental Results and Discussions

**Figure 8:**
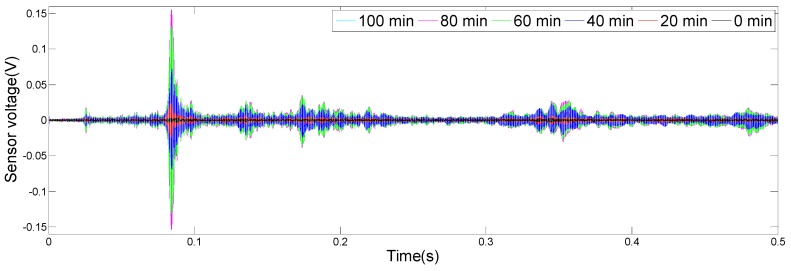
Signal received by PZT1 (0–100 min).

**Figure 9:**
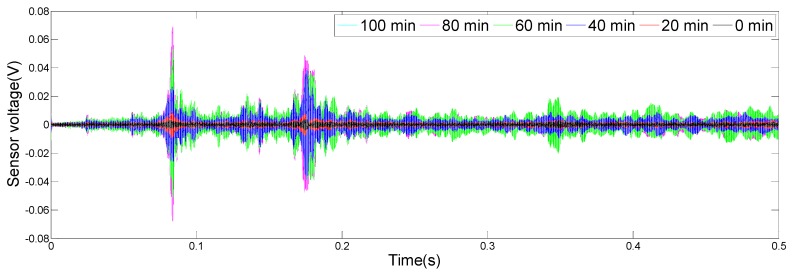
Signal received by PZT2 (0–100 min).

**Figure 10:**
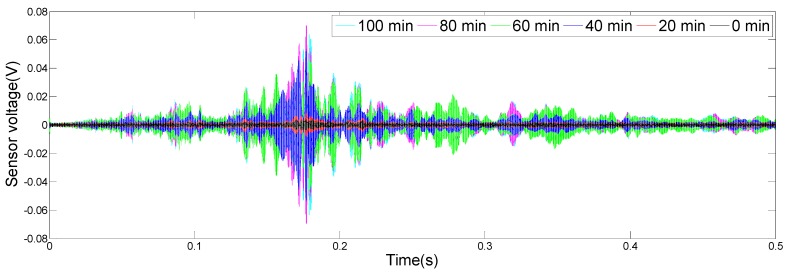
Signal received by SA1 (0–100 min).

**Figure 11:**
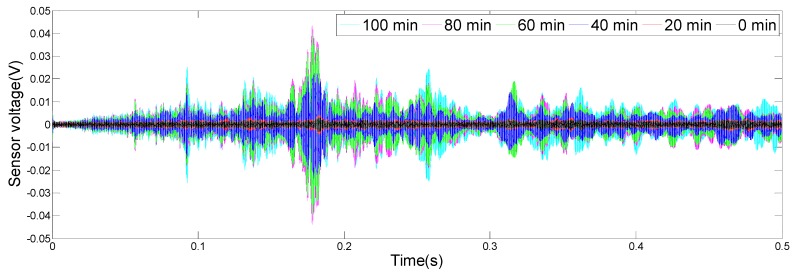
Signal received by SA2 (0–100 min).

The results of the monitoring test are shown in Figure 8, Figure 9, Figure 10 and Figure 11, which show the time domain signal responses that PZT1, PZT2, SA1, and SA2 received at the 0, 20, 40, 60, 80, and 100 min mark, respectively. Each curve represents the signal response of the swept sine wave received by the sensor in 0.5 s. Since the bonding strength between the steel bar and the concrete changes with time during the initial curing period, the signals received by two PZTs and two SAs were different at different time intervals.

It can be seen from Figure 8 that the time domain signal received by PZT1 at the 0th min and the 20th min was close. Afterwards, the time domain signal amplitude increased significantly between 20 and 40 min, similarly, as represented from 40 to 60 min. From 60 to 80 min, the time domain signal amplitude still increased, but at a much a slower rate. In addition, the change in the time domain signal’s amplitude from 80 to 100 min was small. Figure 9, Figure 10 and Figure 11 show that the variation trend of the time domain signals of PZT2, SA1, and SA2 are almost the same as that of PZT1. The results illustrate that the piezoceramic-based active sensing approach can effectively monitor the bonding strength development in real time during the epoxy solidification process.

**Figure 12:**
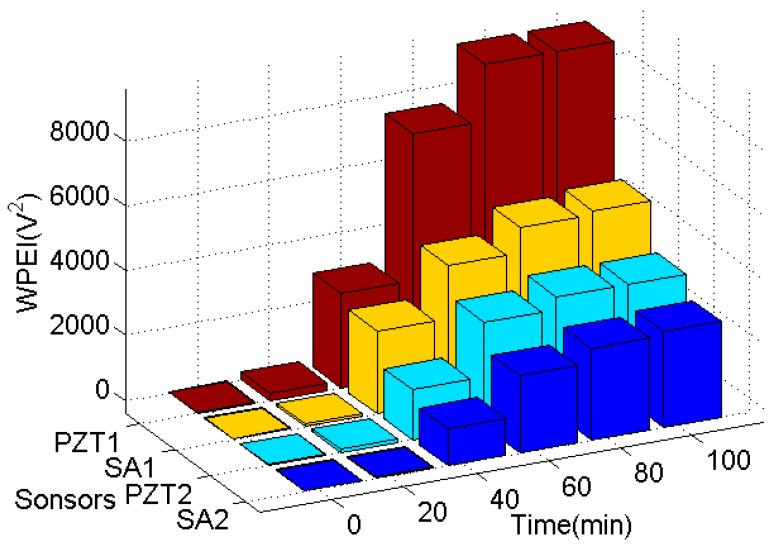
Wavelet-packet-based energy indices (WPEIs) of PZTs and SAs.

Furthermore, the WPEI was applied to quantify the energy of the received signal, as shown in Figure 12. As the energy indices show in this figure, the wave energy was low during the initial 20 min since the epoxy was liquid, and the stress wave can hardly travel through it. A rapid increase in wave energy can be directly observed from 20 to 40 min and from 40 to 60 min for all the PZT sensors and smart aggregates. It suggests that the epoxy begins to solidify and to bond to the steel bar and concrete beam. Therefore, the stress wave can easily transmit from the steel to the concrete beam through the gradually hardened epoxy. From 60 to 80 min, the wave energy increased but at a much slower rate, which suggests that the curing process of the epoxy was close to the end. In addition, the signal energy reached a steady-state value without any obvious changes from 80 to 100 min. It means that the curing process of the epoxy has been completed. The amplitudes of energy indices of PZT1, PZT2, SA1, and SA2 are different because of the different propagation paths of stress waves in the concrete. For example: concrete is not a homogeneous material, and PZT1, PZT2, SA1, and SA2 were deployed in different directions on CB1. Nevertheless, the energy index trends of PZT1, PZT2, SA1, and SA2 are almost the same as the initial 100-min period.

**Figure 13:**
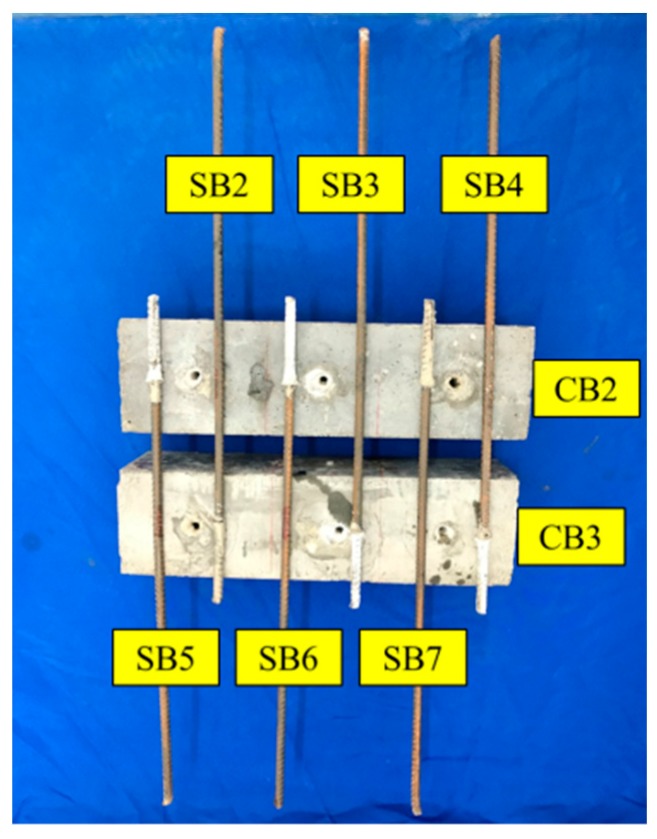
The specimen after the pull-out test.

**Figure 14:**
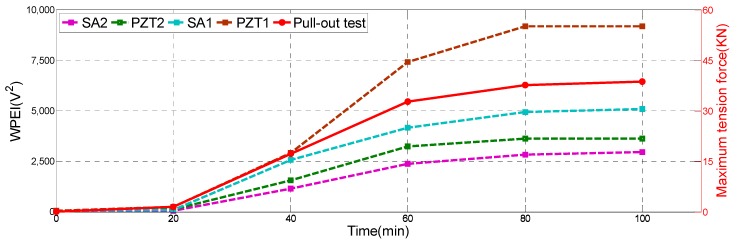
The WPEI and maximum tension value.

Figure 13 shows the specimen after the pull-out test. All steel bars were pulled out from the concrete beams after the test. As shown in Table 2, the maximum tensile force was small and negligible during the initial 20 min, which means that the epoxy has not begun to cure and the corresponding epoxy-grouted bonding strength was very small. The epoxy began to solidify and the bonding strength greatly increased by 31 KN rapidly from 20 to 60 min. From 60 to 80 min, the maximum tensile strength was still increasing, but only increased by 4.93 KN. From 80 to 100 min, the maximum tensile strength also reached a maximum steady-state value to indicate that the solidification process of the epoxy was completed. Comparing the WPEI with the maximum tension in Figure 14, where the four dotted lines represent the energy indices of PZT1, PZT2, SA1, and SA2, and the red solid line represents the maximum tension value of the pull-out test, clearly reveals that the maximum pull-out tensile and WPEI possess a similar trend. The pull-out results also verified the effectiveness of the monitoring results.

**Remark:**  In field implementations, it is important to know when the epoxy reaches its full bonding strength so that the next step of the retrofit or reinforcement, that requires the application of a programmed load through the anchored steel bar, can be carried out. The WPEI results (Figure 12) clearly show that the acquired signal energy reached the maximum value at the 80th min and would not increase after the 100th min. This phenomenon correlates well with the results of the pull-out tests. Basically, from the sensor responses, it is feasible to estimate when the epoxy reaches its full strength, thereby determining the moment of the realization of the planned program of loads for the upgraded structure.

## 5. Conclusions and Future Work

In this paper, a straightforward piezoceramic-based active sensing approach was utilized to monitor epoxy-grouted bonding strength development between an anchored steel bar and the concrete in real time. To verify the proposed methods, experiments were carried out on specimens with anchored steel bars. A PZT patch was bonded to the exposed end of each of the anchored steel bars, and piezoceramic smart aggregates were embedded in the concrete specimens to realize the active sensing approach. In the monitoring test, the time domain signal amplitude and WPEI demonstrate that, during the first 20 min, since the epoxy was liquid, the stress wave can hardly travel through it. During the next 40 min, the epoxy began to solidify and to bond to the steel bar and the concrete beam. Therefore, the stress wave can easily transmit from the steel to the concrete beam through the gradually cured epoxy. During the 60–80 min period, the time domain signal amplitude and WPEI increased but at a much slower rate, which suggests that the curing process of the epoxy was almost completed. In addition, both the received time-domain signals and signal energy reached a steady-state value from 80 to 100 min. It directly indicated that the curing process of the epoxy was completely done. Comparing the WPEI with the maximum tensile force from the pull-out tests clearly shows that the maximum pull-out force and the energy indices are closely correlated. The results of monitoring test and pull-out tests demonstrate that the piezoceramic-based active sensing approach is capable of monitoring the epoxy-grouted bonding strength development in real time.

In further work, the different installation methods of transducers and the sensing range of the PZTs and SAs will be further studied. In addition, aspects of a real-life situation, including external loading and, especially, ambient temperature, will also be investigated. Furthermore, numerical or theoretical models will be developed to compare with the current experimental results.

## Figures and Tables

**Table 1:** The material properties of the test specimens.

Materials	Parameters	Value	Units
Concrete	Density	2400	kg/m^3^
	Young’s modulus	33	Gpa
	Compression strength	40.3	Mpa
Steel bar	Density	7900	kg/m^3^
	Yield strength	350	Mpa
	Tensile strength	530	Mpa
	Elongation	19	%
Epoxy	Tensile strength	14	Mpa
	Compressive strength	65	Mpa
	Flexure strength	53	Mpa
	Bonding strength	17	Mpa
PZT1–PZT3	Dimension	Φ18 × 3	mm
	Piezoelectric strain coefficients (d_33_)	2953.7/2450.4/2151.9	pC/N
SA1–SA2	Dimension	Φ25 × 20	mm
	Piezoelectric strain coefficients (d_33_)	3020.4/2715.9	pC/N

**Table 2:** The maximum tension value of the pull-out test.

	1	2	3	4	5	6
Time (min)	0	20	40	60	80	100
Maximum tension value (KN)	0.12	1.47	17.32	32.81	37.74	38.41

## References

[1] Smith S.T., Hu S., Kim S.J., Seracino R. (2011). FRP-strengthened RC slabs anchored with FRP anchors. Eng. Struct..

[2] Costa R., Providência P., Dias A. (2016). Anchorage Models for Reinforced Concrete Beam-Column Joints under Quasi-Static Loading. ACI Struct. J..

[3] Nanni A. (2003). North American design guidelines for concrete reinforcement and strengthening using FRP: Principles, applications and unresolved issues. Constr. Build. Mater..

[4] Chaallal O., Nollet M. J., Perraton D. (1998). Strengthening of reinforced concrete beams with externally bonded fiber-reinforced-plastic plates: Design guidelines for shear and flexure. Can. J. Civil Eng..

[5] GÓRSKI M., KRZYWOŃ R. (2007). Polish standardisation proposal for design procedures of FRP strengthening. Carbon.

[6] Rocca S., Galati N., Nanni A. (2008). Review of design guidelines for FRP confinement of reinforced concrete columns of noncircular cross sections. J. Compos. Constr..

[7] Biviridge R.L. (1973). Repairs and extensions to concrete structures using resin anchored bars. Civ. Eng. Public Works Rev..

[8] McVay M., Cook R.A., Krishnamurthy K. (1996). Pullout simulation of post installed chemically bonded anchors. J. Struct. Eng..

[9] Mahrenholtz C., Eligehausen R., Reinhardt H.W. (2015). Design of post-installed reinforcing bars as end anchorage or as bonded anchor. Eng. Struct..

[10] Cook R.A. (1992). Load-deflection behavior of cast-in-place and retrofit concrete anchors. Struct. J..

[11] Cook R.A. (1993). Behavior of chemically bonded anchors. J. Struct. Eng..

[12] Cook R.A., Doerr G.T., Klingner R.E. (1993). Bond stress model for design of adhesive anchors. Struct. J..

[13] Cook R.A., Kunz J., Fuchs W., Konz R.C. (1998). Behavior and design of single adhesive anchors under tensile load in uncracked concrete. Struct. J..

[14] Cook R.A., Konz R.C. (2001). Factors influencing bond strength of adhesive anchors. Struct. J..

[15] Li Y., Eligehausen R., Oubolt J., Lehr B. (2002). Numerical analysis of quadruple fastenings with bonded anchors. Struct. J..

[16] Yilmaz S., Özen M.A., Yardim Y. (2013). Tensile behavior of post-installed chemical anchors embedded to low strength concrete. Constr. Build. Mater..

[17] Çalışkan Ö., Yılmaz S., Kaplan H., Kıraç N. (2013). Shear strength of epoxy anchors embedded into low strength concrete. Constr. Build. Mater..

[18] Barnaf J., Bajer M., Vyhnankova M. (2012). Bond strength of chemical anchor in high-strength concrete. Procedia Eng..

[19] Assaad J.J., Issa C.A. (2012). Bond strength of epoxy-coated bars in underwater concrete. Constr. Build. Mater..

[20] Issa C.A., Debs P. (2007). Experimental study of epoxy repairing of cracks in concrete. Constr. Build. Mater..

[21] Aggelis D.G., Shiotani T. (2007). Repair evaluation of concrete cracks using surface and through-transmission wave measurements. Cem. Concr. Compos..

[22] Feng Q., Cui J., Wang Q., Kong Q. (2018). A feasibility study on real-time evaluation of concrete surface crack repairing using embedded piezoceramic transducers. Measurement.

[23] Peng J., Hu S., Zhang J., Cai C.S., Li L.Y. (2019). Influence of cracks on chloride diffusivity in concrete: A five-phase mesoscale model approach. Constr. Build. Mater..

[24] Bajer M., Barnat J. (2012). The glue–concrete interface of bonded anchors. Constr. Build. Mater..

[25] Barnat J., Bajer M. (2015). The shear strength of epoxy adhesive used for chemical anchors. Adv. Mater. Res..

[26] Sakla S.S.S., Ashour A.F. (2005). Prediction of tensile capacity of single adhesive anchors using neural networks. Comput. Struct..

[27] Ryu J.Y., Huynh T.C., Kim J.T. (2019). Tension force estimation in axially loaded members using wearable piezoelectric interface technique. Sensors.

[28] Huynh T.C., Kim J.T. (2016). FOS-based prestress force monitoring and temperature effect estimation in unbonded tendons of PSC girders. J. Aerosp. Eng..

[29] Huo L., Wang F., Li H., Song G. (2017). A fractal contact theory based model for bolted connection looseness monitoring using piezoceramic transducers. Smart Mater. Struct..

[30] Chen B., Hei C., Luo M., Ho M.S., Song G. (2018). Pipeline two-dimensional impact location determination using time of arrival with instant phase (TOAIP) with piezoceramic transducer array. Smart Mater. Struct..

[31] Zhu J., Ho S.C.M., Patil D., Wang N., Hirsch R., Song G. (2017). Underwater pipeline impact localization using piezoceramic transducers. Smart Mater. Struct..

[32] Zou D., Liu T., Qiao G., Huang Y., Li B. (2014). An experimental study on the performance of piezoceramic-based smart aggregate in water environment. IEEE Sens. J..

[33] Moslehy Y., Gu H., Belarbi A., Mo Y.L., Song G. Smart aggregate-based damage detection of circular RC columns under cyclic combined loading. Proceedings of the Structures Congress 2010 on 19th Analysis and Computation Specialty Conference.

[34] Wang D., Zhang J., Zhu H. (2015). Embedded electromechanical impedance and strain sensors for health monitoring of a concrete bridge. Shock Vib..

[35] Müller M., Müller B., Hensel S., Nestler M., Jahn S.F., Müller R., Schubert A., Drossel W.G. (2016). Structural integration of piezoceramic fibers in deep drawn sheet metal for material-integrated health monitoring. Mechatronics.

[36] Song G., Qiao P.Z., Binienda W.K., Zou G.P. (2002). Active vibration damping of composite beam using smart sensors and actuators. J. Aerosp. Eng..

[37] Hu B., Wan J., Pang C.K. (2017). Self-sensing actuation for improved audio-induced vibration rejection in dual-stage hard disk drives. Microsyst. Technol..

[38] Song G., Gu H. (2007). Active vibration suppression of a smart flexible beam using a sliding mode based controller. J. Vib. Control.

[39] Venugopal V.P., Wang G. (2015). Modeling and analysis of Lamb wave propagation in a beam under lead zirconate titanate actuation and sensing. J. Intell. Mater. Syst. Struct..

[40] Agrawal B.N., Elshafei M.A., Song G. (1997). Adaptive antenna shape control using piezoelectric actuators. Acta Astronaut..

[41] Song G., Zhou X., Binienda W. (2003). Thermal deformation compensation of a composite beam using piezoelectric actuators. Smart Mater. Struct..

[42] Wu A., He S., Ren Y., Wang N., Ho S.C.M., Song G. (2019). Design of a New Stress Wave-Based Pulse Position Modulation (PPM) Communication System with Piezoceramic Transducers. Sensors.

[43] Huo L., Li X., Chen D., Li H., Song G. (2017). Identification of the impact direction using the beat signals detected by piezoceramic sensors. Smart Mater. Struct..

[44] Zhu J., Wang N., Ho S.C., Song G. (2018). Method for rapid impact localization for subsea structures. IEEE Sens. J..

[45] Song G., Li H., Gajic B., Zhou W., Chen P., Gu H. (2013). Wind turbine blade health monitoring with piezoceramic-based wireless sensor network. Int. J. Smart Nano Mater..

[46] Feng Q., Kong Q., Song G. (2016). Damage detection of concrete piles subject to typical damage types based on stress wave measurement using embedded smart aggregates transducers. Measurement.

[47] Lu G., Feng Q., Li Y., Wang H., Song G. (2017). Characterization of ultrasound energy diffusion due to small-size damage on an aluminum plate using piezoceramic transducers. Sensors.

[48] Karaiskos G., Flawinne S., Sener J.Y., Deraemaeker A. (2013). Design and validation of embedded piezoelectric transducers for damage detection applications in concrete structures. Key Eng. Mater. Trans. Technol. Publ..

[49] Hu Y., Yang Y. (2007). Wave propagation modeling of the PZT sensing region for structural health monitoring. Smart Mater. Struct..

[50] Liu T., Huang Y., Zou D., Teng J., Li B. (2013). Exploratory study on water seepage monitoring of concrete structures using piezoceramic based smart aggregates. Smart Mater. Struct..

[51] Wang Y., Zhu X., Hao H., Ou J. (2009). Guided wave propagation and spectral element method for debonding damage assessment in RC structures. J. Sound Vib..

[52] Xu K., Deng Q., Cai L., Ho S., Song G. (2018). Damage detection of a concrete column subject to blast loads using embedded piezoceramic transducers. Sensors.

[53] Dao P.B., Staszewski W.J. (2014). Lamb wave based structural damage detection using cointegration and fractal signal processing. Mech. Syst. Signal Process..

[54] Wandowski T., Malinowski P.H., Ostachowicz W.M. (2016). Circular sensing networks for guided waves based structural health monitoring. Mech. Syst. Signal Process..

[55] Żak A., Radzieński M., Krawczuk M., Ostachowicz W. (2012). Damage detection strategies based on propagation of guided elastic waves. Smart Mater. Struct..

[56] Zhang L., Wang C., Song G. (2015). Health status monitoring of cuplock scaffold joint connection based on wavelet packet analysis. Shock Vib..

[57] Mei H., Haider M.F., Joseph R., Migot A., Giurgiutiu V. (2019). Recent Advances in Piezoelectric Wafer Active Sensors for Structural Health Monitoring Applications. Sensors.

[58] Wang F., Huo L., Song G. (2017). A piezoelectric active sensing method for quantitative monitoring of bolt loosening using energy dissipation caused by tangential damping based on the fractal contact theory. Smart Mater. Struct..

[59] Kong Q., Song G. (2017). A comparative study of the very early age cement hydration monitoring using compressive and shear mode smart aggregates. IEEE Sens. J..

[60] Xu J., Wang C., Li H., Zhang C., Hao J., Fan S. (2018). Health monitoring of bolted spherical joint connection based on active sensing technique using piezoceramic transducers. Sensors.

[61] Zhang J., Li Y., Huang Y., Jiang J., Ho S.C. (2018). A feasibility study on timber moisture monitoring using piezoceramic transducer-enabled active sensing. Sensors.

[62] Huynh T.C., Lee S.Y., Dang N.L., Kim J.T. (2019). Sensing Region Characteristics of Smart Piezoelectric Interface for Damage Monitoring in Plate-Like Structures. Sensors.

[63] Wang F., Ho S.C.M., Huo L., Song G. (2018). A novel fractal contact-electromechanical impedance model for quantitative monitoring of bolted joint looseness. IEEE Access.

[64] Fan S., Li W., Kong Q., Feng Q., Song G. (2018). Monitoring of pin connection loosening using eletromechanical impedance: Numerical simulation with experimental verification. J. Intell. Mater. Syst. Struct..

[65] Zhang J., Zhang C., Xiao J., Jiang J. (2019). A PZT-Based Electromechanical Impedance Method for Monitoring the Soil Freeze–Thaw Process. Sensors.

[66] Shi Y., Luo M., Li W., Song G. (2018). Grout compactness monitoring of concrete-filled fiber-reinforced polymer tube using electromechanical impedance. Smart Mater. Struct..

[67] Feng Q., Kong Q., Jiang J., Liang Y., Song G. (2017). Detection of interfacial debonding in a rubber–steel-layered structure using active sensing enabled by embedded piezoceramic transducers. Sensors.

[68] Jiang T., Kong Q., Wang W., Huo L., Song G. (2016). Monitoring of grouting compactness in a post-tensioning tendon duct using piezoceramic transducers. Sensors.

[69] Wang T., Song G., Wang Z., Li Y. (2013). Proof-of-concept study of monitoring bolt connection status using a piezoelectric based active sensing method. Smart Mater. Struct..

[70] Kong Q., Fan S., Bai X., Mo Y.L., Song G. (2017). A novel embeddable spherical smart aggregate for structural health monitoring: Part I. Fabrication and electrical characterization. Smart Mater. Struct..

[71] Qin F., Kong Q., Li M., Mo Y.L., Song G., Fan F. (2015). Bond slip detection of steel plate and concrete beams using smart aggregates. Smart Mater. Struct..

[72] Zeng L., Parvasi S.M., Kong Q., Huo L., Li M., Song G. (2015). Bond slip detection of concrete-encased composite structure using shear wave based active sensing approach. Smart Mater. Struct..

[73] Kong Q., Robert R., Silva P., Mo Y. (2016). Cyclic crack monitoring of a reinforced concrete column under simulated pseudo-dynamic loading using piezoceramic-based smart aggregates. Appl. Sci..

[74] Xu K., Ren C., Deng Q., Jin Q., Chen X. (2018). Real-time monitoring of bond slip between GFRP bar and concrete structure using piezoceramic transducer-enabled active sensing. Sensors.

[75] Li W., Fan S., Ho S.C.M., Wu J., Song G. (2018). Interfacial debonding detection in fiber-reinforced polymer rebar–reinforced concrete using electro-mechanical impedance technique. Struct. Health Monit..

[76] Di B., Wang J., Li H., Zheng J., Zheng Y., Song G. (2019). Investigation of Bonding Behavior of FRP and Steel Bars in Self-Compacting Concrete Structures Using Acoustic Emission Method. Sensors.

[77] Xu Y., Luo M., Hei C., Song G. (2018). Quantitative evaluation of compactness of concrete-filled fiber-reinforced polymer tubes using piezoceramic transducers and time difference of arrival. Smart Mater. Struct..

[78] Zhang J., Li Y., Du G., Song G. (2018). Damage detection of L-shaped concrete filled steel tube (L-CFST) columns under cyclic loading using embedded piezoceramic transducers. Sensors.

[79] Wu J., Kong Q., Li W., Song G. (2017). Interlayer slide detection using piezoceramic smart aggregates based on active sensing approach. IEEE Sens. J..

[80] Kong Q., Hou S., Ji Q., Mo Y.L., Song G. (2013). Very early age concrete hydration characterization monitoring using piezoceramic based smart aggregates. Smart Mater. Struct..

[81] Kong Q., Wang R., Song G., Yang Z.J., Still B. (2014). Monitoring the soil freeze-thaw process using piezoceramic-based smart aggregate. J. Cold Reg. Eng..

[82] Asgarian B., Aghaeidoost V., Shokrgozar H.R. (2016). Damage detection of jacket type offshore platforms using rate of signal energy using wavelet packet transform. Mar. Struct..

[83] Lei D., Yang L., Xu W., Zhang P., Huang Z. (2017). Experimental study on alarming of concrete micro-crack initiation based on wavelet packet analysis. Constr. Build. Mater..

